# Efficacy and safety of pembrolizumab with preoperative neoadjuvant chemotherapy in patients with resectable locally advanced head and neck squamous cell carcinomas

**DOI:** 10.3389/fimmu.2023.1189752

**Published:** 2023-07-31

**Authors:** Kai Wang, Lin Gui, Haizhen Lu, Xiaohui He, Dezhi Li, Chang Liu, Shaoyan Liu, Xiaolei Wang

**Affiliations:** ^1^ Department of Head and Neck Surgery, National Cancer Center/National Clinical Research Center for Cancer/Cancer Hospital, Chinese Academy of Medical Sciences and Peking Union Medical College, Beijing, China; ^2^ Department of Medical Oncology, National Cancer Center/National Clinical Research Center for Cancer/Cancer Hospital, Chinese Academy of Medical Sciences and Peking Union Medical College, Beijing, China; ^3^ Department of Pathology, National Cancer Center/National Clinical Research Center for Cancer/Cancer Hospital, Chinese Academy of Medical Sciences and Peking Union Medical College, Beijing, China; ^4^ Department of Positron Emission Tomography/Computer Tomography (PET/CT) Center, National Cancer Center/National Clinical Research Center for Cancer/Cancer Hospital, Chinese Academy of Medical Sciences and Peking Union Medical College, Beijing, China

**Keywords:** Head and neck carcinoma, neoadjuvant, immunotherapy, pembrolizumab, laryngeal function preservation

## Abstract

**Background:**

This study aimed to explore the efficacy and safety of pembrolizumab combined with chemotherapy as neoadjuvant therapy in patients with resectable locally advanced head and neck squamous cell carcinomas (LA-HNSCCs).

**Methods:**

In this prospective, single-arm, single-centre clinical trial, patients meeting the inclusion criteria were treated with preoperative neoadjuvant therapy with 200 mg pembrolizumab combined with 75 mg/m2 cisplatin and 175 mg/m2 paclitaxel. This was followed by surgery and postoperative adjuvant therapy. The primary endpoint was the postoperative pathological complete response (pCR) rate. All statistical analyses were performed using SPSS 26.

**Results:**

A total of 22 patients were enrolled. The location of primary lesion showed: hypopharynx were 15 (68.2%), oropharynx were 6 (27.3%) and oral cavity was 1 (4.5%). The postoperative pCR rate, was 36.4% (8/22), and there was no delay to surgery due to adverse drug reactions. The rate of laryngeal function preservation was 90.9% (20/22). Delayed wound healing was the main surgical complication, with an incidence of 22.7% (5/22). The median follow-up time was 9.5 months, and only 1 patient (4.55%) suffered a regional recurrence.

**Conclusion:**

Preoperative treatment with pembrolizumab and chemotherapy in resectable LA-HNSCC has a high pCR rate with no significant impact on surgical safety. This treatment was found to increase the rate of laryngeal function preservation. However, the effects of neoadjuvant immunotherapy on long-term prognosis in LA-HNSCCs require further study.

## Research background

Over 60% of head and neck squamous cell carcinomas (HNSCCs) were diagnosed at advanced stage, which raises therapeutic challenge and results in poor prognosis (1, 2). The functional anatomy of the head and neck is complex and important. Either as a direct consequence of the tumour invasion or because of the tumour treatment, patients with LA-HNSCC often suffer from damage to important functions such as respiration, swallowing and speech pronunciation, with significant deleterious effects on their quality of life. Therefore, there is an urgent need to improve both the therapeutic efficacy and the function preservation of LA-HNSCC treatment.

In recent years, the value of immune checkpoint inhibitors, especially anti-programmed cell death-1 (PD-1) and anti-programmed cell death ligand-1 (PD-L1) monoclonal antibodies in the treatment of recurrent/metastatic HNSCCs has received increasing recognition among the academic community. Pembrolizumab is a PD-1 blocker that minimises the inhibitory effects of PD-1 on T cells, thereby enhancing the anti-tumour action of T cells. A phase III clinical trial (KeyNote-048 study) compared treatment with pembrolizumab, both alone and in combination with chemotherapy, versus the EXTREME regimen (combination therapy consisting of a platinum agent, 5-fluorouracil and cetuximab) for recurrent/metastatic HNSCCs. Both alone and in combination with chemotherapy, pembrolizumab was found to be significantly superior to cetuximab combined with chemotherapy and to effectively prolong overall survival (OS) (3). Based on the above results, pembrolizumab is recommended as a first-line treatment for recurrent/metastatic HNSCCs.

The efficacy of immune checkpoint inhibitors promises improved outcomes in the treatment of HNSCCs. The lower tumour heterogeneity, fewer drug-resistant clones and better immune status of patients before surgery makes them more responsive to neoadjuvant immunotherapy, with higher compliance than recurrent/metastatic patients (4). Preliminary results (5–10) have been published from current trials of preoperative immune checkpoint inhibitor and chemotherapy treatment, but these studies focus on pathological downstaging and postoperative pathological responses. The contribution of neoadjuvant immunotherapy to functional preservation during surgery has not previously been reported.

Neoadjuvant immunotherapy is known to improve the postoperative pathological response in a proportion of HNSCC patients. Our previous trial (11) showed that oral cavity cancer patients who achieved pathologic complete response (pCR) following induction chemotherapy had a prolonged survival. Therefore, we speculate that neoadjuvant immunotherapy is likely to have both survival benefits and functional preservation benefits. Thus, this clinical study investigated the effects of preoperative treatment with pembrolizumab and neoadjuvant chemotherapy with cisplatin and paclitaxel in patients with resectable LA-HNSCCs. This paper is a report of our preliminary results.

## Materials and methods

### Enrolled patients

This was a prospective, single-arm, single-centre clinical trial (registration no.: ChiCTR2200055719). Patients recently treated for HNSCCs at the National Cancer Centre/Cancer Hospital of the Chinese Academy of Medical Sciences between April 2021 and June 2022 were enrolled. The inclusion criteria were aged ≥18 y with histopathologically confirmed squamous cell carcinomas and locally advanced cases of P16-positive oropharyngeal carcinoma at clinical stage II-III, or non-P16-positive oropharyngeal carcinoma and other HNSCCs at stage III-IV suitable for complete surgical resection. The exclusion criteria were previous treatment with PD-1 monoclonal antibodies or similar drugs, radical surgical resection not possible, distant metastasis and allergies to the drugs used in this study. The study protocol was approved by the Ethics Committee of the National Cancer Centre/Cancer Hospital of the Chinese Academy of Medical Sciences (approval no.: 21/056-2727). All enrolled patients gave written informed consent to trial participation.

### Research process

The patients were treated with pembrolizumab 200 mg combined with cisplatin 75 mg/m^2^ and paclitaxel 175 mg/m^2^ as neoadjuvant therapy. The drugs were administered on the first day of a three-week treatment cycle. Surgery was performed about 4 weeks after the last neoadjuvant treatment. Regardless of any lesion regression after neoadjuvant therapy, the scope of surgical resection was determined according to the baseline pre-treatment assessment. Postoperative adjuvant therapy was determined by a multidisciplinary team (MDT) based on pathological high-risk factors and the preoperative clinical stage of the patient.

Prior to drug treatment and again before surgery, the patients were examined with contrast-enhanced computed tomography of the head, neck and chest to assess the range of their lesions. The response to neoadjuvant therapy was evaluated using the RECIST 1.1 standard. The safety of the neoadjuvant therapy was determined by recording any adverse reactions within 30 d of receiving treatment. These were graded using the Common Terminology Criteria for Adverse Events (CTCAE 5.0). Surgery was defined as delayed if it was not performed within 7 weeks of the last neoadjuvant therapy. Postoperative pathology was evaluated based on the residual tumours in the resected specimens. Based on other solid tumors and KeyNote-689 research design (12–14), pCR was defined as no residual tumour tissue in either the primary lesion or metastatic lymph nodes. The pCR of lymph nodes and primary lesions were established separately. A major pathological response (MPR) was defined as <10% residual tumour of the primary lesion. The expression of PD-1 in primary lesions before neoadjuvant therapy and in residual lesions after surgery was described using the combined positive score (CPS) of 22C3 in immunohistochemical staining to explore any correlation between PD-1 expression and the efficacy of neoadjuvant immunotherapy. CPS is a measure of the ratio of PD-L1 positive cells to total tumour cells.

### Data analysis

All statistical analyses were performed using SPSS for Windows version 26 (IBM Corp., Armonk, NY, USA). Categorical variables were described as frequencies (percentages) and analysed with two-tailed Fisher’s exact tests. Continuous variables were analysed with two-sample exact Mann–Whitney–Wilcoxon rank sum tests. P-values <0.05 were considered statistically significant. All risk factors found to be significant in univariate analyses were included in multivariate logistic regression analysis.

## Results

### Enrolment and baseline characteristics of patients

**Figure 1:**
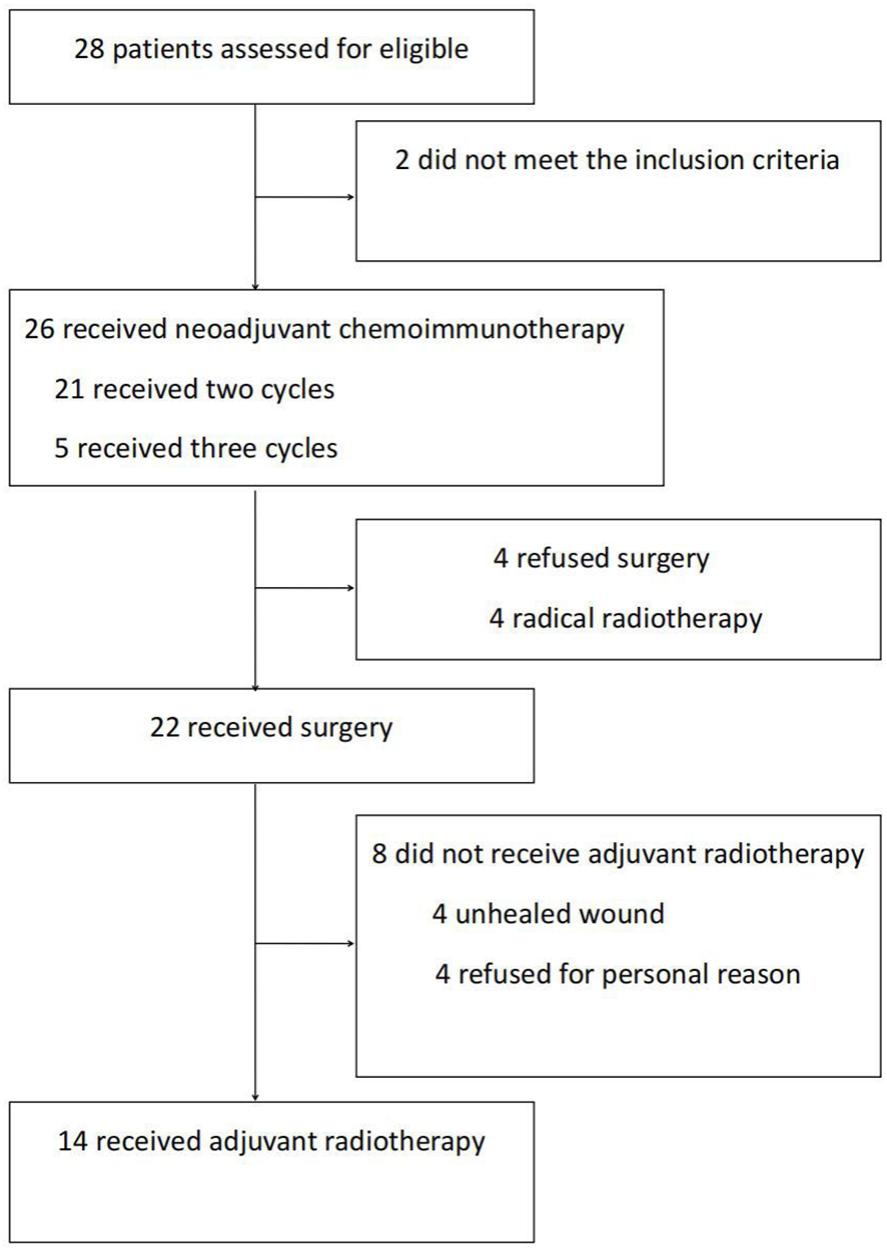
Trail flow diagram.

Between April 2021 and June 2022, our research group recruited 28 patients to participate in this trial. Among them, 2 patients did not meet the inclusion criteria. The remaining 26 received neoadjuvant immunotherapy. After therapy, 4 patients refused surgery and chose radiotherapy instead. Finally, 22 patients who received neoadjuvant therapy followed by surgery were included in our data analyses (**Figure 1**). This comprised 21 men and 1 woman (mean age, 58.1 y). The primary lesions were predominantly classified as hypopharyngeal carcinomas (n = 15), followed by oropharyngeal carcinomas (n = 6) and oral carcinomas (n = 1). Before treatment, the T-stage of most of the primary lesions was T3–4 (n = 14). Of the patients with hypopharyngeal carcinomas, 9 suffered from postcricoid region involvement accompanied by unilateral vocal cord fixation and 2 from oesophageal entrance involvement. None had bilateral vocal cord involvement. The most common initial symptom of the patients was cervical lymph node enlargement. Before treatment, the majority of the patients (n = 19) were in lymph node category N2, with patients’ largest metastatic lymph node having an average diameter of 2.74 ± 1.15 cm. The largest metastatic lymph node of 9 patients was >3 cm. The above characteristics indicate late local T and N category in this patient group, and most lesions were at clinical stage IV (n = 16). The 3 patients at clinical stage II were all diagnosed with P16-positive oropharyngeal carcinomas at T- category T2–3 and N- category N2 (**Table 1**).

**Table 1:** Patients’ demographics features and clinicopathological characteristics before surgery.

Variables	Number of patients	%
Male/Female	21/1	
Age (mean ± SD, years)	58.1 ± 10.49(22~69)	–
Primary site		
Oropharynx	6	27.3
hypopharynx	15	68.2
Oral Cavity	1	4.5
Hypopharynx invasion(N=15)		
Fixed vocal cord	9	60
Extend to esophagus	2	13.3
Tobacco		
YES	16	72.7
NO	6	27.3
Alcohol		
YES	15	68.2
NO	7	31.8
P16 states		
Positive	4	18.2
Negative	18	81.8
T stage		
T1-T2	8	36.4
T3	7	31.8
T4	7	31.8
N stage		
N0-N1	3	13.6
N2	19	86.4
Stage		
II	3	13.6
III	3	13.6
IV	16	72.7
PD-L1 combined positive score		
<5	7	31.8
5-10	2	9.1
≥10	13	59.1

### Safety and clinical efficacy evaluations

Among the enrolled patients, 17 underwent two cycles of pembrolizumab combined with chemotherapy, and 5 underwent three cycles. These 5 patients received an additional cycle because their surgery was delayed by the COVID-19 epidemic. None of the patients had delayed surgery due to adverse drug reactions. The safety evaluation of pembrolizumab combined with preoperative neoadjuvant chemotherapy recorded 2 patients with grade 3 adverse reactions, both of which were leukopenia caused by bone marrow suppression and no grade 4 adverse reactions. The most common grade 1 or 2 adverse reactions were nausea and anorexia (n = 6), followed by rashes (n = 3) and weakness (n = 3) (**Table 2**). Our efficacy evaluation found regression of primary lesions or metastatic cervical lymph nodes after neoadjuvant therapy, with partial remission in 18 patients and stable disease in 4 patients. No disease progression (PD) or hyperprogression occurred during treatment.

**Table 2:** Treatment-related adverse events (TRAEs).

EVENTS	Patients(N=22)		
	Grade1-2(%)	Grade 3(%)	Grade4(%)
Nausea/vomiting	6 (27.3)	0	0
Rash	3 (13.6)	0	0
Fatigue	3 (13.6)	0	0
Leukopenia	2 (9.1)	2 (9.1)	0
Anemia	2 (9.1)	0	0
Neurotoxicity	1 (4.5)	0	0
Diarrhea	1 (4.5)	0	0
Increased aminotransferases	1 (4.5)	0	0
Hypertension	1 (4.5)	0	0
Alopecia	1 (4.5)	0	0

### Surgery and relevant pathological evaluation

The patients underwent surgery about 4 weeks after their final neoadjuvant treatment, with a mean interval of 34.9 d. 8 of the 15 patients with hypopharyngeal carcinomas underwent a pyriform sinus resection or posterior pharyngeal wall resection, 5 underwent a partial laryngectomy and hypopharyngectomy, and 2 underwent a total laryngectomy and hypopharyngectomy. The overall rate of laryngeal preservation was 90.9% (20/22). Among the 7 patients with oropharyngeal carcinomas and oral carcinomas, partial tongue or tongue root resection was performed in 3, partial laryngectomy and tongue root resection in 2 and tonsillectomy in 2 patients. In 12 patients, the primary lesion was closed directly; in 8, it was reconstructed with adjacent flaps; and in 2, it was reconstructed with free flaps. Unilateral cervical lymph node dissection was performed in 10 patients, and bilateral cervical lymph node dissection in 12 patients. Radical unilateral cervical lymph node dissection was only performed in 1 patient, with the rest receiving modified radical dissections. Delayed wound healing was the main postoperative complication, with an incidence of 22.7% (5/22). There were no severe perioperative complications or perioperative deaths. All postoperative complications were cured by conservative treatment.

**Figure 2:**
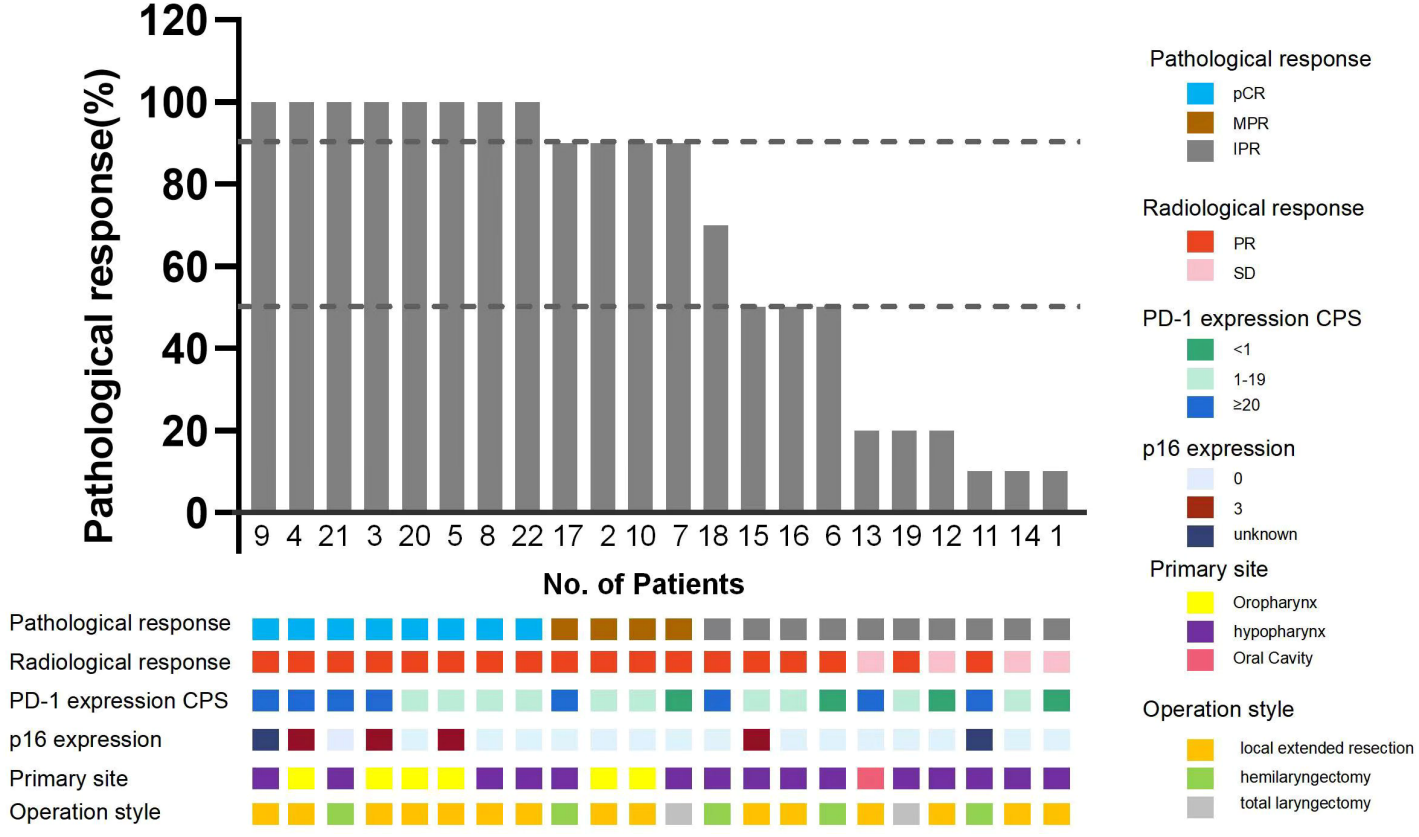
Pathological response of the enrolled patients. pCR, Pathological complete response; MPR, Major pathological response; IPR, Incomplete pathological response; p16 expression 0 for negative, 3 for strong positive.

Eight patients achieved pCR (36.4%). In the remaining 14 patients without pCR, residuals were found in both the primary lesions and the cervical lymph nodes in 5 patients; 1 patient had a pCR only in the primary lesion, and 8 had pCR only in the lymph nodes. The overall cervical lymph node pCR rate was 71.4% (15/21) (cN0 (clinical lymph node negative), n = 1), and the overall pCR rate for primary lesions was 40.6% (9/22). Among the 13 patients with residual primary lesions, 1 presented with only local carcinoma *in situ* and 6 with scattered lesions with shallow depth of invasion (DOI) and severe atypical hyperplasia around it. The rate of MPR was 54.5% (12/22), and 17 patients had a lower pathologic stage, only one patient with a pathology confirmed prevertebral fascia invade reclassified the T4b vs. T2 in pre-surgery. In the 9 patients with hypopharyngeal carcinomas accompanied by unilateral vocal cord fixation, the MPR rate was also high (55.6%, 5/9). Only 2 patients with hypopharyngeal carcinomas underwent total laryngectomies (**Figure 2**). Postoperative pathological high-risk factors included vascular tumour thrombi (n = 2) and extranodal extensions (n = 2). All surgeries were R0 resections, and postoperative pathology examinations found no positive margins. Three of the 22 patients had residuals at the first intraoperative incisal margin. After extensive resection, no residual tumour was found at the incisal margins.

To identify variables associated with pCR, we performed correlation analyses between pCR and patients’ demographic and clinical characteristics, condition and disease stage before neoadjuvant therapy. CPS and P16 status were used as markers for statistical analyses. There were 15 (68.2%) patients with CPS >5 in the biopsies of their primary lesions, and this was found to be correlated with pCR (*p* = 0.015). pCR was also correlated with the pre-treatment tumour stage (*p* = 0.028). We found no correlations between primary lesions and the diameter of the cervical lymph nodes, the diameter of primary lesions or P16 status (**Table 3**).

**Table 3:** Correlation Analysis for Pathological Complete Response.

	Pathological Complete Response		P		Pathological Complete Response		P
	YES	NO			YES	NO	
**Age**, years	56.4 ± 7.5	59.0 ± 12.0	0.585	**N stage**			
**Gender**				N0-N1	0	3 (21.4)	0.159
Male	7 (87.5)	14 (100)	0.176	N2	8 (100)	11 (78.6)	
Female	1 (12.5)	0		**Stage**			
**Primary site**				II	3 (37.5)	0	0.028*
Oropharynx	0	1 (7.1)	0.168	III	0	3 (21.4)	
Hypopharynx	4 (50)	11 (78.6)		IV	5 (62.5)	11 (78.6)	
Oral Cavity	4 (50)	2 (14.3)		**CPS>5**			
**Tobacco**				YES	8 (100)	7 (50)	0.015*
YES	4 (50)	12 (85.7)	0.070	NO	0	7 (50)	
NO	4 (50)	2 (14.3)		**CPS>10**			
**Alcohol**				YES	7 (50)	6 (21.4)	0.040*
YES	4 (50)	11 (78.6)	0.166	NO	1 (50)	8 (78.6)	
NO	4 (50)	3 (21.4)		**Radiographic evaluation**			
**P16 states**				PR	8 (100)	10 (71.4)	0.095
Positive	3 (37.5)	1 (7.1)	0.076	SD	0	4 (28.6)	
Negative	5 (62.5)	13 (92.9)		**Primary site diameter**	1.9 ± 0.97	2.5 ± 1.1	0.267
**T stage**							
T1-T2	4 (50)	4 (28.6)	0.325	**LN diameter**	3.2 ± 0.6	2.5 ± 1.3	0.098
T3	3 (37.5)	4 (28.6)					
T4	1 (12.5)	6 (42.9)		**Time interval for surgery**	37.9 ± 7.8	33.2 ± 10.3	0.281

PR, Partial response; SD, Stable disease; CPS, Combined Positive Score; LN, Lymph nodes.

### Adjuvant radiotherapy/chemoradiotherapy

In this trial, 14 patients received adjuvant radiotherapy/chemoradiotherapy after surgery, while 4 did not undergo radiotherapy due to the presence of unhealed wounds after surgery, and 4 more with postoperative pCR did not undergo radiotherapy based on comprehensive multidisciplinary team discussions and the patients’ wishes. 5 patients received adjuvant chemoradiotherapy due to the presence of IPR and risk factor of distant metastasis, other 9 patients received adjuvant radiotherapy. Adjuvant radiotherapy/chemoradiotherapy was given at 4-6 weeks after surgery, only one patient had an early termination of adjuvant radiotherapy due to infection of COVID-19 during radiotherapy.

### Follow-up

**Figure 3:**
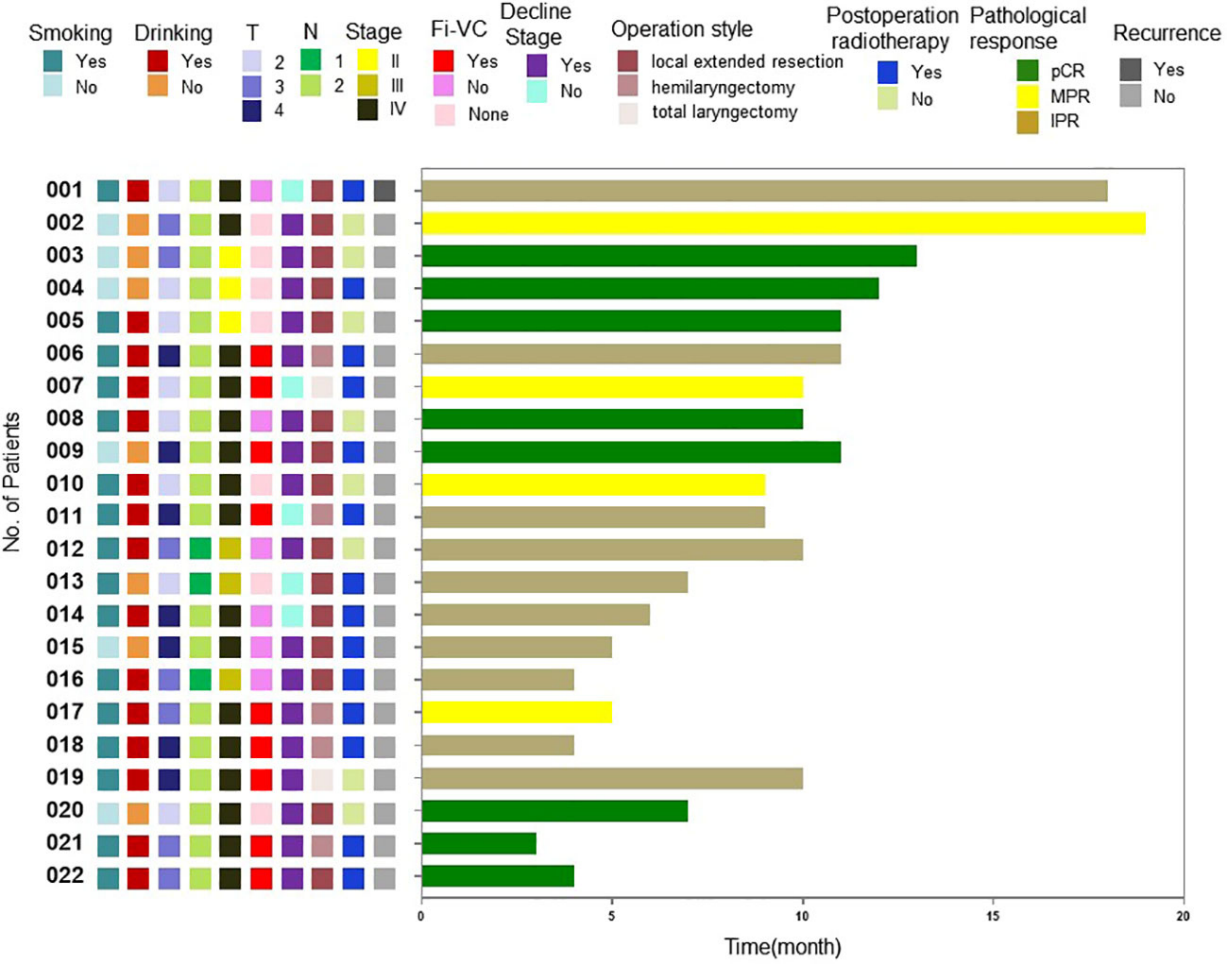
Swimming plot of disease-free survival for individual patients.Fi-VC, Fixed vocal cord; pCR, Pathological complete response; MPR, Major pathological response; IPR, Incomplete pathological response.

At the time of writing, the longest follow-up time has been 19 months, with a median follow-up time of 9.5 months. At present, observation of all patients is ongoing, and 1 patient suffered a recurrence of regional lymph node during the follow-up. The recurrence case had a surgical pathology showed an incomplete post-treatment pathological response, and there were >90% clinical residual lesions. After surgery, chemoradiotherapy was performed. Local-regional lymph node lesions recurred 6 months after follow-up. These were treated with chemotherapy, resulting in a one-year disease-free survival rate of 95.5%. The long-term therapeutic effects and the correlations between pathological response rates and patients’ condition before treatment and therapeutic effects require validation by further follow-up data (**Figure 3**).

## Discussion

The response rate to neoadjuvant therapy of the patients in our sample was high. The postoperative rate of pCR after surgical resection was 36.4% (8/22). Zinner et al. treated 26 patients with preoperative nivolumab combined with carboplatin and paclitaxel as neoadjuvant therapy and reported a pCR rate of 42% (5), while a recent study of preoperative camrelizumab combined with chemotherapy reported a pCR rate of 37% (7). Our pCR rates were roughly concordant with those of previous reports. Current data in the literature indicate that pCR rates from immunotherapy combined with chemotherapy are higher than those from immunomonotherapy or dual-drug immunotherapy combined with a neoadjuvant regimen (8, 9, 15). When combined with immunotherapy, chemotherapy can have synergistic effects on the induction of immunogenic cell death, the up-regulation of tumour antigen expression and disruption of the immunosuppressive tumour microenvironment (16).

After classification and analysis of the postoperative pathological examinations of the primary lesions and cervical lymph node lesions, we found that, of the 13 patients with postoperative residual lesions, only 1 had only residual lymph node tumour tissue with no residual primary lesion; 7 patients had residual primary lesions, but lymph nodes that were pathologically negative for metastatic tumour tissue and 5 patients had residual malignancy in both the primary lesion and cervical lymph nodes. The responses of the primary lesions and cervical lymph nodes to pembrolizumab combined with neoadjuvant chemotherapy were not synchronous, with lymph nodes showing a higher response rate than primary lesions. The CIAO study also reported a better response to immune checkpoint inhibitors in metastatic lymph nodes than primary lesions (4). Although the specific mechanism behind this is unclear, previous research on neoadjuvant chemotherapy for breast cancer has similarly found that metastatic lymph nodes are more likely to achieve a pCR than primary lesions. A pCR in metastatic lymph nodes also showed a higher correlation with improved long-term prognoses in the breast cancer study (17).

CPS is the most widely used marker of the effects of anti-PD-1 drug therapy (18). We found a correlation between pre-treatment CPS levels >5 and pCR, suggesting that CPS also has predictive significance for the effects of pembrolizumab combined with neoadjuvant chemotherapy and immunotherapy. However, previous evaluations of the clinical applicability of CPS have produced differing results. The KEYNOTE-048 study found that patients with CPS between 1 and 20 benefit more from immunotherapy (3), while the CHECKMATE-141 study found that CPS between 5 and 10 were not correlated with positive immunotherapy outcomes (19). This discrepancy warrants further study. Previous research has tested other molecular markers, including tumour mutation burden (TMB), CD8+TiL and IL-6, but the prediction of pCR after neoadjuvant immunotherapy remains poor (20).

Pembrolizumab combined with neoadjuvant chemotherapy provides favourable conditions for functional preservation during surgery. Many of the patients in this study had hypopharyngeal carcinomas and tongue-base carcinomas adjacent to the larynx. However, the overall rate of laryngeal function preservation was 90.9% (20/22). In the past, patients with hypopharyngeal carcinomas at T3 and above accompanied by unilateral vocal cord fixation were usually treated with total laryngectomy (21). Of the 9 patients with postcricoid region involvement accompanied by unilateral vocal cord fixation in this study, only 2 underwent total laryngectomy, while the others were treated with partial laryngectomies and hypopharyngeal resections to achieve radical resection with R0. Previously, platinum-based induction chemotherapy improved the laryngeal function preservation rate but did not affect OS (22). However, the purpose of induction chemotherapy is to improve the proportion of functional preservation through the selection of appropriate concurrent chemoradiotherapy. In contrast, neoadjuvant immunotherapy aims to achieve functional preservation by improving the proportion of partial laryngectomy. Most of the patients in our sample had metastatic lymph nodes with a diameter >3 cm, which were shrunk to varying degrees by neoadjuvant therapy. Only 1 patient underwent radical lymph node dissection; in the rest, the lymph nodes were treated with modified radical dissection, avoiding the functional damage caused by radical dissection.

In terms of surgical safety, no complications that affected surgery resulted from the neoadjuvant therapy. Nevertheless, immunotherapy is not without adverse effects, which include bone marrow suppression, nausea, rashes and hypothyroidism (8). No disease progression occurred in any of the patients after neoadjuvant immunotherapy. However, in previous immunotherapy trials, there have been instances of false progression in imaging-based RECIST assessments after treatment due to the inflammatory infiltration caused by regional immune activation of local lesions (23). During surgery, a variety of repair methods were attempted, including local skin flaps, free jejunum flaps and free skin flaps. There were no skin flap-related complications in our patients. Compared with preoperative radiotherapy, pembrolizumab combined with neoadjuvant chemotherapy has less influence on the selection of surgical repair methods.

Despite its advantages, pembrolizumab combined with neoadjuvant chemotherapy also presents challenges in surgical treatment. The most common postoperative surgical complications of the patients treated with neoadjuvant therapy were poor wound healing, infection around the tracheal stoma, pharyngeal fistula and lymphatic fistula. Most recovered from these complications with conservative treatment, but postoperative radiotherapy was delayed in several patients because of complications. In addition, it was found during surgery that 3 of the 22 patients had residuals at the first intraoperative incisal margin. Most of the 12 patients with residual lesions also had scattered lesions with shallow DOI (depth of invasion) accompanied by severe peripheral atypical hyperplasia. This suggests that, despite observable lesion regression after neoadjuvant therapy, residual microscopic lesions and atypical hyperplasia in the area of the primary lesion often remain. Therefore, in patients with apparent lesion regression, narrowing of the surgical scope is inadvisable. It is also necessary to standardise the safety boundary reservation and the submission of intraoperative incisal margins to prevent residuals. The problems with incisal margins have received little attention in previous studies on neoadjuvant therapy. This is largely because most previous research has used patients with oral and oropharyngeal carcinomas. In our study, hypopharyngeal carcinomas accounted for the vast majority of the patients, so safety-boundary-related problems were prominent.

At follow-up, all 22 patients were in stable condition. The longest follow-up time was 19 months. One patient suffered local-regional lymph node recurrence. Long-term outcomes will become apparent with further postoperative follow-up. At present, the 3-year recurrence rate of patients with advanced HNSCCs is >50% (2), and the 5-year OS rates of patients at stage III and IVA are 61% and 32%, respectively (24). Whether neoadjuvant therapy affects long-term survival remains to be seen. In most instances, locally advanced HNSCCs usually requires adjuvant radiotherapy after primary surgery. Although many of our patients had negative pathology results and tumour downstaging after surgery, 14 still chose to undergo radiotherapy. Postoperative radiotherapy is an important adjuvant treatment. Given the poor prognosis of advanced HNSCCs, especially hypopharyngeal carcinomas, high treatment intensity is conducive to tumour control. A recent study of the anti-PD-1 immune checkpoint inhibitor camrelizumab combined with neoadjuvant chemotherapy reported a case of postoperative pCR without local recurrence after radiotherapy and death after 14 months, indicating that postoperative adjuvant radiotherapy remains important (7). However, in the CIAO study of simultaneous immunotherapy and dual-drug immunotherapy, 45% of patients received neoadjuvant immunotherapy and surgery only, avoiding the adverse effects of post-oropharyngeal surgery radiotherapy on swallowing, mucosa production, mouth opening and other functions and improving their quality of life (4).

Analysis of the reasons for previous treatment failures in HNSCCs, represented by hypopharyngeal carcinomas, showed that multiple primary mucosal lesions due to physicochemical factors, submucosal invasion and high and occult metastasis of cervical lymph nodes were the main causes of local failure and early and rapid distant metastasis significantly affects long-term survival after surgery. Neoadjuvant therapy has the potential to improve long-term tumour immunity through immune memory. In addition, the response rate to preoperative neoadjuvant therapy was higher than that from anti-recurrence/metastasis treatment. This is likely because of the lower tumour heterogeneity, fewer drug-resistant clones and better immune status of the patients. It is conceivable that neoadjuvant therapy might be more effective to overcome the aforementioned adverse factors in HNSCC treatment and provide long-term tumour control and longer survival.

## Data availability statement

The original contributions presented in the study are included in the article/supplementary material. Further inquiries can be directed to the corresponding author.

## Ethics statement

This study was approved by the Ethics Committee Cancer Center/Cancer Hospital, Chinese Academy of Medical Sciences (approval No.: 21/056-2727). The patients/participants provided their written informed consent to participate in this study.

## Author contributions

KW: data curation, software, formal analysis, validation, investigation, methodology, writing-original draft, writing-review and editing. LG: resources, data curation, investigation, methodology, writing-original draft. HL: funding acquisition, resources, data curation, investigation, methodology, writing-original draft. XH: supervision, investigation. DL: resources, data curation. CL: formal analysis, validation, investigation, methodology, writing-original draft, writing-review. SL: supervision, investigation. XW: conceptualization, resources, data curation, supervision, validation, investigation, methodology, writing-original draft, project administration, writing-review and editing. All authors discussed the results and contributed to the final manuscript. All authors contributed to the article and approved the submitted version.
